# Prediction of Seropositivity in Suspected Autoimmune Encephalitis by Use of Radiomics: A Radiological Proof-of-Concept Study

**DOI:** 10.3390/diagnostics14111070

**Published:** 2024-05-21

**Authors:** Jacob Stake, Christine Spiekers, Burak Han Akkurt, Walter Heindel, Tobias Brix, Manoj Mannil, Manfred Musigmann

**Affiliations:** 1 University Clinic for Radiology, University of Münster and University Hospital Münster, Albert-Schweitzer-Campus 1, 48149 Münster, Germany; jacob.stake@uni-muenster.de (J.S.);; 2 Institute of Medical Informatics, University of Münster, Albert-Schweitzer-Campus 1, 48149 Münster, Germany

**Keywords:** autoimmune encephalitis, seropositivity, radiomics, MRI, machine learning, automated machine learning (AutoML), neuroimaging, artificial intelligence

## Abstract

In this study, we sought to evaluate the capabilities of radiomics and machine learning in predicting seropositivity in patients with suspected autoimmune encephalitis (AE) from MR images obtained at symptom onset. In 83 patients diagnosed with AE between 2011 and 2022, manual bilateral segmentation of the amygdala was performed on pre-contrast T2 images using 3D Slicer open-source software. Our sample of 83 patients contained 43 seropositive and 40 seronegative AE cases. Images were obtained at our tertiary care center and at various secondary care centers in North Rhine-Westphalia, Germany. The sample was randomly split into training data and independent test data. A total of 107 radiomic features were extracted from bilateral regions of interest (ROIs). Automated machine learning (AutoML) was used to identify the most promising machine learning algorithms. Feature selection was performed using recursive feature elimination (RFE) and based on the determination of the most important features. Selected features were used to train various machine learning algorithms on 100 different data partitions. Performance was subsequently evaluated on independent test data. Our radiomics approach was able to predict the presence of autoantibodies in the independent test samples with a mean AUC of 0.90, a mean accuracy of 0.83, a mean sensitivity of 0.84 and a mean specificity of 0.82, with Lasso regression models yielding the most promising results. These results indicate that radiomics-based machine learning could be a promising tool in predicting the presence of autoantibodies in suspected AE patients. Given the implications of seropositivity for definitive diagnosis of suspected AE cases, this may expedite diagnostic workup even before results from specialized laboratory testing can be obtained. Furthermore, in conjunction with recent publications, our results indicate that characterization of AE subtypes by use of radiomics may become possible in the future, potentially allowing physicians to tailor treatment in the spirit of personalized medicine even before laboratory workup is completed.

## 1. Introduction

Autoimmune encephalitis (AE) comprises a group of non-infectious neurological disorders caused by a misdirected immune response against self-antigens of the central nervous system. While believed to be extremely rare in the past, recent studies suggest that AE incidence nearly matches that of infectious encephalitis [1]. Clinical manifestation is often subacute and includes seizures, short-term memory loss, altered mental status, and psychiatric symptoms [2]. Due to a lack of large, randomized trials, first-line therapy is mostly empirical and entails corticosteroids, intravenous immunoglobulin (IVIG), and plasma exchange (PE) [3]. As early treatment has been shown to improve the outcome, rapid diagnostic workup is vital [4,5].

Even though uniform criteria for diagnosing AE have been proposed [2], diagnosis is often delayed due to the non-specificity of symptoms and heavy reliance on laboratory testing for antineuronal antibodies. In the last decades, a variety of these autoantibodies have been identified, enabling differentiation of various AE subtypes. Depending on antigen localization, antibodies can be divided into two groups.

Antibodies against intracellular antigens such as anti-Hu and anti-Ma are generally associated with limited response to first line immunotherapy and frequently occur concomitant to an underlying neoplasm [6]. Associated tumors include small cell lung cancer, testicular tumors, and breast cancer [7]. These antibodies are not pathogenic, but rather appear concurrent with a cytotoxic T-cell reaction causing neuronal damage [8,9,10].

In contrast, antibodies targeting cell surface proteins such as anti-NMDAR and anti-AMPAR have been shown to directly suppress neuronal function in a titer-dependent manner [11]. As this effect is often reversible under immunotherapy, prognosis is generally better [12,13]. Prevalence of accompanying malignancies varies in these AE subtypes, with anti-NMDAR encephalitis showing the strongest association with ovarian teratoma [6,12].

In about half of all AE cases, no antineuronal antibodies can be detected [14]. These cases, referred to as antibody AE or seronegative AE, rely on clinical criteria for diagnosis [2].

**Figure 1:**
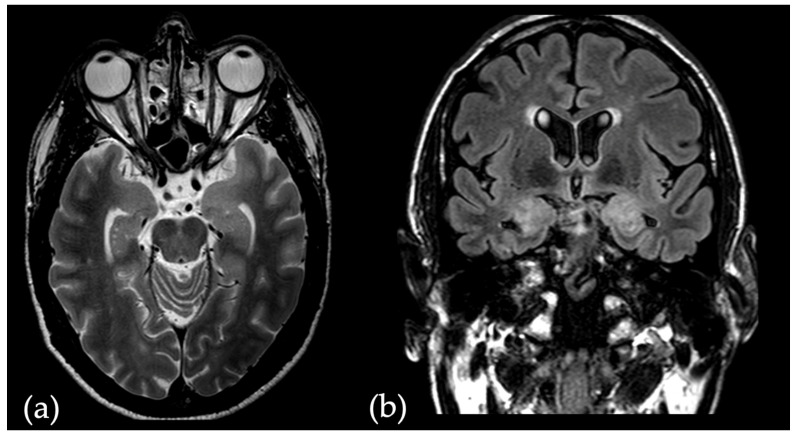
(**a**) T2-image of a 54-year-old seropositive AE patient who presented with serial seizures. Left-leading hyperintensity and enlargement of the mesial temporal lobe. (**b**) Corresponding fluid attenuated inversion recovery (FLAIR) sequence in the coronal plane showing bilateral hyperintensity of the mesial temporal lobe.

Given the implications of seropositivity for definitive diagnosis of suspected AE cases, we sought to develop an ML-backed radiomics model capable of differentiating AE patients with confirmed antibody presence and seronegative patients. Because Magnetic Resonance Imaging (MRI) at symptom onset frequently shows uni- or bilateral involvement of the mesial temporal lobe (e.g., Figure 1) [15], we chose to train our classifier model on radiomic features obtained from manual bilateral segmentation of the amygdala on pre-contrast T2 images. This is in line with histopathological studies finding lymphocytic infiltration and neuronal damage located in the mesial temporal lobe in AE patients [16,17].

The radiomics approach utilizes the extraction and subsequent analysis of various quantitative features from regions of interest (ROIs) in medical images. Commonly used open-source feature libraries such as PyRadiomics include an abundance of pre-defined radiomic features, resulting in high-dimensional sets of mineable data that are difficult to analyze using conventional statistical approaches [18]. With recent advances in artificial intelligence research, machine learning models can be used to link imaging data and clinical endpoints by identifying complex radiological patterns otherwise not perceptible to the human eye [19].

For example, recent studies have shown radiomics’ potential in differentiating demyelinating diseases of the central nervous system [20], identifying Parkinson’s disease subtypes [21] and discriminating grade II glioma from brain inflammation [22].

Application to AE yielded promising results in predicting the prognosis of anti-NMDAR encephalitis [23], differentiating AE from Herpes simplex encephalitis [24], and discerning AE and low-grade diffuse astrocytoma [25].

Given these favorable results showing radiomics’ potential in differential diagnosis and prognosis prediction in AE, we sought to apply the radiomics approach to our cohort of AE patients. Because detection of antineuronal antibodies still plays an important role in confirming suspected AE cases, we aimed to explore the non-invasive prediction of seropositivity by building an ML-backed classifier model. This approach could potentially be of interest for expediting diagnostic workup, especially in institutions with limited resources for laboratory testing. With recent studies suggesting antibody-specific structural and metabolic correlates in the mesial temporal lobe [26,27], we chose to train our model on radiomics features obtained from bilateral segmentation of the amygdala.

## 2. Materials and Methods

The study was performed in compliance with the Declaration of Helsinki [28] and approved by the local ethics committee (Ärztekammer Westfalen-Lippe (ÄKWL) Münster 2022-298-f-S). Due to its retrospective nature, written informed consent was waived. Compliance with national and state data protection laws was assured. Our aim was to determine seropositivity in patients with autoimmune encephalitis using machine learning. We used a binary differentiation. This means that we intended to apply machine learning to distinguish between patients with previously detected and undetected AE-specific autoantibodies. For this purpose, we identified AE patients who presented to our hospital between August 2011 and February 2022 by performing an ICD-10 code-based retrospective query of our patient database. Patient records were screened, and cohorts were assessed according to the diagnosis and antibody status listed in the respective discharge letters. Our analyses were based on (native) T2-weighted MRI images obtained at symptom onset in which manual bilateral segmentation of the amygdala was performed. Imaging data were acquired using 1.5T and 3T scanners at our institution and various secondary care centers. Image preprocessing was performed to assure comparability (see below). Segmentation of the region of interest was performed in the axial plane. MRI images obtained at symptom onset were available for 101 patients. We excluded 18 patients from the study due to nonavailability of (native) T2-weighted MR images (5), insufficient imaging quality (11), or neuroanatomical variations (e.g., hippocampal malrotation (2)). Antibodies were detected in 43 of the remaining 83 patients, and correspondingly no antibodies were detected in 40 cases. The demographic characteristics of our study cohort are summarized in Table 1.

### 2.1. Radiomics

We performed the bilateral segmentation of the amygdala using the 3D Slicer software (version 4.10, www.slicer.org, accessed on 20 April 2024). Manual segmentation was performed in the axial plane. Anatomical reference points used are detailed in the segmentation protocol by Moore et al. [29]. The single-reader segmentation was reviewed by the above mentioned experienced neuroradiologist. Standardized preprocessing steps were performed on all images to assure comparability. Spatial resampling to 2 × 2 × 2 voxels was performed and a uniform bin width of 64 was set. Subsequently, 107 radiomic features were extracted from the regions of interest of each patient’s T2-weighted MRI images using the PyRadiomics software (https://pyradiomics.readthedocs.io/en/latest/, accessed on 20 April 2024), available as an implementable plugin for the 3D Slicer platform. We used radiomic features of the classes “First Order Statistics”, “Shape-based”, “Gray Level Co-occurrence Matrix”, “Gray Level Run Length Matrix”, “Gray Level Size Zone Matrix”, “Neighboring Gray Tone Difference Matrix”, and “Gray Level Dependence Matrix”. In addition to these radiomic features, we also used the age of the patients at the time of the MRI examination and their gender as further possible features. To exclude redundant information, all features were subjected to a 95% correlation filter.

### 2.2. Statistical Analysis

Statistical analysis was performed using R software (version 4.1.3). The 83 patients in the final study cohort were randomly assigned to a training group and an independent test group, using a stratified 4:1 ratio with a balanced distribution of patients with detected and undetected antibodies between these two groups (compare Table 1). Feature preselection and subsequent model development, including hyperparameter tuning, were performed using the training data (i.e., 80% of the total data). The subsequent determination of the model performance was carried out with independent test data (the remaining 20% of the total data). To identify the most promising machine learning algorithm classes, we started our analyses using automated machine learning (AutoML) from H_2_O. H_2_O AutoML simultaneously optimizes various decision tree-based models, Gradient Boosting Machines (GBMs), XGBoost GBMs, Deep Neural Nets (DNNs), and Generalized Linear Models (GLMs). For more information about the included models, see the H_2_O AutoML documentation [30]. In addition, H_2_O AutoML uses combinations of the basic algorithms listed, so-called “Stacked Ensemble Models (SEMs)”. Since we primarily used H_2_O AutoML to initially identify the most promising algorithm classes for the clinical problem we analyzed, we did not use SEMs. In these initial analyses, we found that GLM algorithms and neural networks are the most promising. Good, but comparatively lower, discriminatory power could also be achieved with GBMs. Accordingly, we subsequently tuned a Lasso (Least absolute shrinkage and selection operator) regression, a ridge regression, an elastic net, a neural net, and a GBM. Hyperparameters included in the models were optimized using 10-fold cross-validation. The models themselves were optimized by maximizing the area under the curve (AUC) of the receiver operator characteristic (ROC).

Performance was determined in terms of AUC, accuracy, sensitivity, specificity, positive predictive value (PPV), and negative predictive value (NPV) using independent test data (i.e., the remaining 20% of the total data). Accuracy describes the proportion of correctly predicted cases overall, sensitivity the proportion of correctly predicted cases with detected antibodies, and specificity the proportion of correctly predicted cases without detected antibodies. Finally, PPV describes the proportion of correctly predicted cases with detected antibodies in relation to all predicted cases with detected antibodies, and NPV is the corresponding ratio for non-detected antibodies.

The model performance achieved depends on both the feature preselection and the number of features contained in the models. We therefore used two alternative approaches to determine the features and their optimal number. On the one hand, we used recursive feature elimination (RFE), based on random forest (see, for example, Darst et al. [31]). On the other hand, we developed all models with an increasing number of features, each containing only the most important 1 to 15 features. Starting with a one-feature model, the most important features were included in each model with a predefined number of features. These features were determined beforehand using the “varImp”-function in R (varImp = variable importance). This function calculates the performance gain achieved by each individual feature and thus determines the most important features in each case. In this second approach to preselect the features, the optimal number of model features to be included was determined by analyzing at which model complexity (in terms of the number of features included) the highest model performance was achieved. This approach minimizes the risk of possible overfitting.

**Figure 2:**
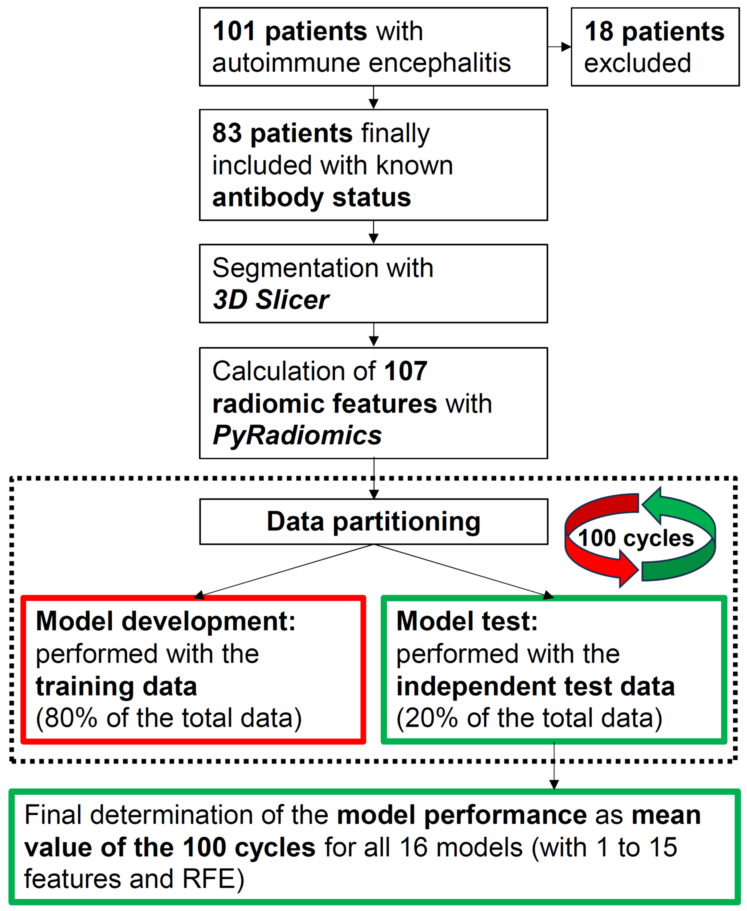
Flowchart describing the methodological approach. For each tested machine learning algorithm (i.e., GBM, neural network, Lasso regression, ridge regression, and an elastic net), a total of 16 models was developed, including an increasing number (1 to 15) of model features on the one hand and the features determined using RFE (recursive feature elimination) on the other. We developed each of our models 100 times (100 cycles). For each of these 100 cycles, a new data partitioning was carried out and the associated model was subsequently tested with independent test data. The final model performance was finally calculated as the average value of the previous 100 cycles, based on the independent test data used in each cycle.

Finally, since the model performance achieved also depends on the partitioning of the data into training and independent test data, we performed the data partitioning and subsequent complete model development and testing for each model using 100 different data partitions. All performance values were always calculated as the mean values of these 100 runs. In addition, we determined the 95% confidence intervals. To facilitate understanding, the entire process is illustrated in a flow chart (see Figure 2). Reference is also made to Musigmann et al. [32], where the exact procedure is described in more detail.

## 3. Results for Predicting Seropositivity in Suspected AE Using Machine Learning

As already explained, we used two different methods to preselect the features to be included in our models: Firstly, recursive feature elimination and secondly, we developed all models with an increasing number of features, each containing the most important 1 to 15 features. In the final modeling phase, we pursued those machine learning algorithms that proved most promising based on the preliminary studies previously conducted with AutoML. GLMs (Lasso regression, ridge regression, and an elastic net) and neural networks followed by GBMs proved to be particularly promising for discriminating autoimmune encephalitis patients with detected and undetected antibodies.

**Figure 3:**
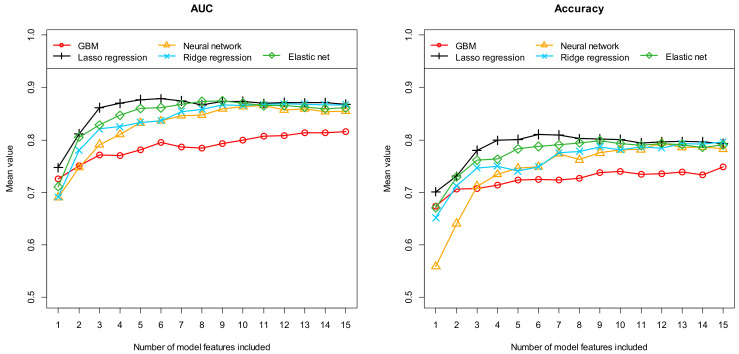
Prediction of seropositivity (detectable/no detectable antibodies) in patients with suspected autoimmune encephalitis: Area under the curve (AUC) and accuracy for the independent test samples, calculated as means of 100 repetitions (100 cycles) depending on the number of model features included. Five different machine learning algorithms (as indicated in the figures) were tested for feature preselection and subsequent model construction. Each individual curve describes the discriminatory power achieved with the corresponding machine learning algorithm as a function of the number of features included.

**Figure 4:**
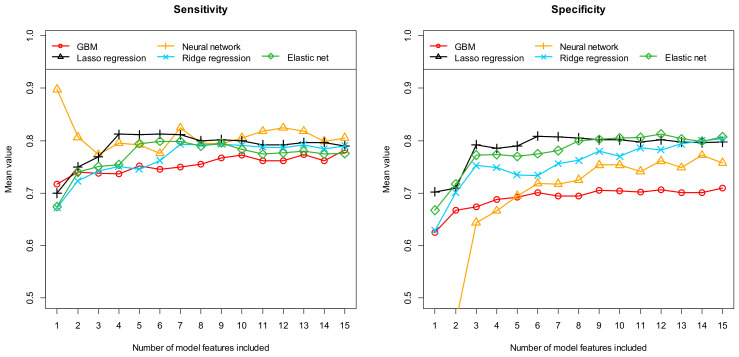
Prediction of seropositivity (detectable/no detectable antibodies) in patients with suspected autoimmune encephalitis: Sensitivity and specificity for the independent test samples, calculated as means of 100 repetitions (100 cycles) depending on the number of model features included. Five different machine learning algorithms (as indicated in the figures) were tested for feature preselection and subsequent model construction. Each individual curve describes the discriminatory power achieved with the corresponding machine learning algorithm as a function of the number of features included.

**Figure 5:**
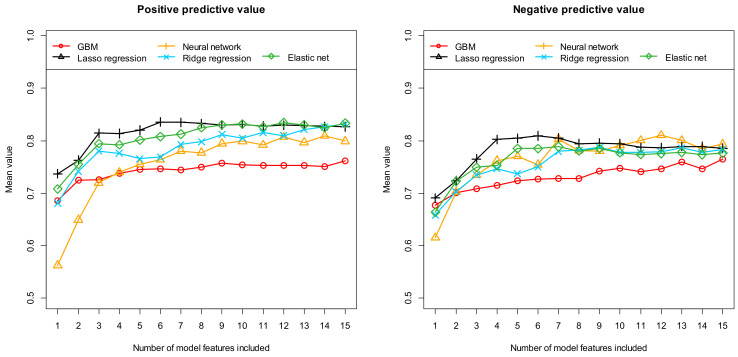
Prediction of seropositivity (detectable/no detectable antibodies) in patients with suspected autoimmune encephalitis. The positive predictive value and the negative predictive value were calculated as averages of 100 cycles based on independent test samples (see text). Five different machine learning algorithms (as indicated in the figures) were tested for feature preselection and subsequent model construction. Each individual curve describes the discriminatory power achieved with the corresponding machine learning algorithm as a function of the number of features included.

Firstly, Table 2 summarizes the results obtained with these five different machine learning algorithms after performing feature preselection using RFE. Figure 2, Figure 3 and Figure 4, on the other hand, show the results obtained with the most important 1 to 15 features. Here, the performance results achieved in terms of AUC and accuracy are summarized in Figure 3, in terms of sensitivity and specificity in Figure 4, and finally in terms of the positive and negative predictive values in Figure 5. As previously explained, all results were determined based on independent (unknown) test data and as mean values of 100 complete runs (each run consisting of a complete model development and subsequent testing). The models achieve their highest performance in the range between 6 and 12 features. If more features are used, the performance does not increase any further or even begins to decrease slightly. This characterizes the beginning of an overfitting. On average, 22.1 and 17 features are selected when using RFE (mean number and median, respectively). Models with this number of features are already slightly overfitted, which in turn implies that the models developed with RFE perform slightly worse on average than the best models developed with our alternative feature preselection method.

Comparing the results achieved with the five different machine learning algorithms, the GBMs performed worst on average, which had already been indicated in the preliminary test using AutoML. The results obtained with the ridge regression, the elastic net, and the neural network are usually between the results of the GBM and the Lasso regression. Overall, the Lasso regression models, including six final features, perform best. On average over 100 runs, the six-feature Lasso regression models exhibit an AUC of 0.878 [0.653, 1.000], an accuracy of 0.811 [0.588, 1.000], a sensitivity of 0.812 [0.444, 1.000], a specificity of 0.809 [0.566, 1.000], a PPV of 0.836 [0.613, 1.000], and a NPV of 0.809 [0.564, 1.000]. The numbers in brackets indicate the 95% confidence interval.

As we have described, all models were fully developed 100 times each, using 100 different data partitions. Accordingly, the 100 resulting models can differ in their feature composition. Therefore, we analyzed how different these 100 models really are. For each feature included in at least one of the six-feature models, we determined the number of runs in which this feature was selected. For the 10 most frequently selected features, Table 3 lists the number of 100 runs in which the respective feature was selected.

The two features “firstorder_Minimum” and “shape_Flatness” are included in almost all models (96 of 100 runs). The four further features are included in 82% of the models (“gldm_DependenceNonUniformity”), in 73% of the models (“firstorder_Kurtosis”), in 68% of the models (“glszm_GrayLevelNonUniformity”), and finally in 59% of the models (“glcm_Idn”). Beyond these six most frequently selected features, the importance of the other possible additional features decreases sharply. This again fits with the fact that the discriminatory power of the Lasso regression models no longer increases from the sixth feature onwards (compare Figure 3, Figure 4 and Figure 5). In order to determine the extent to which the performance achieved by our models depends on the exact individual model composition, we additionally developed a model that contains the six most frequently selected features of the 100 runs (see Table 3) in fixed form. This model was again developed using Lasso regression and 100 different data partitions. This time, however, the features contained in the models were fixed during the 100 runs. The classification results of the six-feature models are summarized in Table 4 for the case of a variable feature composition on the one hand (column “Different features”), and for this fixed feature composition on the other hand (column “Fixed features”). Our methodology with 100 repetitions is well suited to analysis of the stability of a particular model approach and comparison of the different approaches. The approach we chose provided very stable results. The models developed with the different features exhibit very similar discriminatory power to the models developed subsequently with fixed features. In detail, the two approaches (different features/fixed features) using six features resulted in the following mean performance values: mean AUC = 87.8%/90.2%, mean accuracy = 81.1%/83.1%, mean sensitivity = 81.2%/84.0%, mean specificity = 80.9%/82.0%, mean PPV = 83.6%/84.9%, and mean NPV = 80.9%/83.4%. Thus, our models show high performance in differentiating patients with and without detectable autoantibodies, i.e., in predicting seropositivity in suspected AE cases.

## 4. Discussion

Based on the assumption that medical imaging contains exploitable information reflecting underlying pathophysiology, the radiomics approach utilizes the extraction and subsequent analysis of various standardized radiomic features from radiological images. In recent years, radiomics has shown promising potential beyond its original application in oncology imaging [20,21,33,34]. However, application to AE has been less extensively studied. To the best of our knowledge, only three studies applying the radiomics approach to MR images from AE patients have been published.

Promising results have been achieved in distinguishing AE cases from patients with Herpes simplex encephalitis and normal controls using a combination of radiomic features obtained from segmentation of the hippocampus and deep learning [24], predicting prognosis of anti-NMDAR encephalitis by training a random forest classifier on hippocampal radiomic features [23], and differentiating AE from low-grade diffuse astrocytoma by building a joint ML-model utilizing radiomic and spatial distribution features [25].

Furthermore, a machine learning approach utilizing data on mesial temporal lobe volumetry was successfully used to differentiate AE cases with LGI1 and GAD autoantibodies, suggesting antibody-specific structural correlates in the mesial temporal lobe [26]. In another study, application of a deep learning approach to positron emission tomography (PET) images showed favorable results in differentiating AE patients with either LGI1 or GABA-B autoantibodies, indicating antibody-specific metabolism changes in the mesial temporal lobe [27].

Early diagnosis of AE still poses a significant challenge. Although clinical criteria have been proposed to reduce reliance on laboratory testing [2], detection of AE-specific antibodies is still widely used to confirm the diagnosis, possibly delaying treatment before confirmatory results from send-out testing can be acquired. Our radiomics approach was able to predict seropositivity in our cohort of suspected AE patients with a mean AUC of 0.90, a mean accuracy of 0.83, a mean sensitivity of 0.84, and a mean specificity of 0.82 in the independent test samples. The six radiomic features included in our final model entail shape-based (shape_flatness), first-order (e.g., histogram-based: firstorder_Minimum, firstorder_Kurtosis), and second-order (e.g., texture-based: gldm_DependenceNonUniformity, glszm_GrayLevelNonUniformity, glcm_Idn) features. Thus, our model is mainly reliant on pattern changes in voxel intensity in the amygdalae, with features characterizing intensity homogeneity (gldm_DependenceNonUniformity, glszm_GrayLevelNonUniformity, glcm_Idn) playing an important role. This allows for careful interpretation that seropositivity-dependent voxel intensity alterations in the amygdala provide discriminatory power that can be harnessed by the radiomics approach. These results indicate that radiomics may become a helpful tool for non-invasive prediction of antibody presence in suspected AE cases in the future.

Furthermore, our findings in conjunction with the above-mentioned recent publications suggest that further characterization of AE subtypes by use of radiomics may become possible soon. Because AE subtypes differ in terms of optimal diagnostic workup, treatment approach, and possible paraneoplastic etiology, this may enable physicians to tailor treatment and diagnostic resources in the spirit of personalized medicine even before laboratory test results can be obtained. Given the difference in prognosis and likelihood of paraneoplastic etiology [6], radiomics-based differentiation between AE cases with onconeural antibodies against intracellular antigens and antibodies targeting cell surface antigens may be of particular interest. However, larger cohorts are needed to explore this approach.

### 4.1. Limitations

Several limitations of this study need to be addressed. Due to its retrospective character, inherent limitations were present. Moreover, a total of 18 patients had to be excluded due to nonavailability of (native) T2-weighted MR images (5), insufficient imaging quality (11) or neuroanatomical variations (e.g., hippocampal malrotation (2)). Furthermore, the subjective character of manual ROI segmentation and risk of inherent bias need to be addressed. Additionally, due to the rarity of AE cases, our unseen test samples were rather small. Larger prospective cohorts are required to validate our results. Finally, we acknowledge that due to the approach used to screen our patient database, overinterpretation of our seronegative patient cohort is possible. Furthermore, due to the possibility of false-positive antibody test results, possible overestimation of the clinical significance of seropositivity needs to be addressed. Despite these limitations, our ML-backed approach yielded promising results on previously unseen datasets in independent testing.

### 4.2. Conclusions

In conclusion, our results indicate that radiomics could be a promising tool for non-invasively predicting seropositivity in suspected AE patients. Given the implications of seropositivity for definitive diagnosis of suspected AE cases, this may expedite diagnostic workup even before results from laboratory testing can be obtained. Especially on sites with poor infrastructure for specialized laboratory testing, this may be beneficial, as potential seropositivity could be assessed before definitive results from send-out testing sites can be obtained.

However, larger, prospective studies are needed to confirm our findings before translation into clinical practice may become possible.

## Figures and Tables

**Table 1:** Demographic characteristics of our cohort with suspected autoimmune encephalitis to predict seropositivity (detectable/no detectable antibodies). All data calculated as means of 100 repetitions (100 cycles, see below).

	Training Data	Independent Test Data	Total Data
Number of patients (*n*)	66	17	83
Gender (in %): Female/Male	50.55/49.45	50.82/49.18	50.60/49.40
Mean age (in years)	50.79	51.68	51.38
Antibody detected (in %): Yes/No	51.52/48.48	52.94/47.06	51.81/48.19
Antibody detected (number): Yes/No	34/32	9/8	43/40

**Table 2:** Prediction of seropositivity (detectable/no detectable antibodies) in patients with suspected autoimmune encephalitis using RFE (recursive feature elimination) to preselect the model features. All results calculated as means of 100 repetitions (100 cycles) using independent test data. AUC: area under the curve, PPV: positive predictive value and NPV: negative predictive value.

Performance Metric	Model Algorithm				
	GBM	Neural Network	Lasso Regression	Ridge Regression	Elastic Net
AUC	0.808 [0.521:0.993]	0.845 [0.556:1.000]	0.863 [0.625:1.000]	0.852 [0.539:1.000]	0.850 [0.585:1.000]
Accuracy	0.740 [0.529:0.941]	0.784 [0.588:1.000]	0.793 [0.501:0.941]	0.785 [0.529:1.000]	0.782 [0.560:0.941]
Sensitivity	0.771 [0.392:1.000]	0.804 [0.503:1.000]	0.802 [0.392:1.000]	0.773 [0.444:1.000]	0.772 [0.444:1.000]
Specificity	0.705 [0.375:1.000]	0.761 [0.441:1.000]	0.783 [0.500:1.000]	0.799 [0.500:1.000]	0.793 [0.500:1.000]
PPV	0.756 [0.559:1.000]	0.804 [0.613:1.000]	0.811 [0.529:1.000]	0.819 [0.586:1.000]	0.813 [0.586:1.000]
NPV	0.750 [0.500:1.000]	0.792 [0.545:1.000]	0.797 [0.482:1.000]	0.774 [0.500:1.000]	0.773 [0.524:1.000]

**Table 3:** Most important features for predicting seropositivity (detectable/no detectable antibodies) using (native) T2-weighted MRI images. Calculations are based on the six-feature models developed using Lasso regression.

Level of Importance	Feature Name	Number of Runs Included
1	firstorder_Minimum	96
2	shape_Flatness	96
3	gldm_DependenceNonUniformity	82
4	firstorder_Kurtosis	73
5	glszm_GrayLevelNonUniformity	68
6	glcm_Idn	59
7	glrlm_RunLengthNonUniformity	15
8	glszm_SizeZoneNonUniformityNormalized	12
9	glcm_InverseVariance	11
10	gldm_DependenceNonUniformityNormalized	11

**Table 4:** Classification results for predicting seropositivity (detectable/no detectable antibodies) for the six-feature Lasso regression models. The features included in the models were, on the one hand, newly determined in each of the 100 cycles (“Different features”) and, on the other hand, fixed according to Table 3 (“Fixed features”). The positive predictive value and the negative predictive value were calculated as averages of 100 cycles based on independent test samples (see text). All performance metrics were calculated as averages of 100 cycles based on independent test samples. The 95% confidence intervals are shown in parentheses.

Performance Metric	Different Features	Fixed Features
AUC	0.878 [0.653:1.000]	0.902 [0.757:1.000]
Accuracy	0.811 [0.588:1.000]	0.831 [0.619:1.000]
Sensitivity	0.812 [0.444:1.000]	0.840 [0.444:1.000]
Specificity	0.809 [0.566:1.000]	0.820 [0.625:1.000]
PPV	0.836 [0.613:1.000]	0.849 [0.684:1.000]
NPV	0.809 [0.564:1.000]	0.834 [0.586:1.000]

## Data Availability

Data available on request. The data presented in this study are available on reasonable request from the corresponding author.
